# Karyotype Diversity, Mode, and Tempo of the Chromosomal Evolution of Attina (Formicidae: Myrmicinae: Attini): Is There an Upper Limit to Chromosome Number?

**DOI:** 10.3390/insects12121084

**Published:** 2021-12-02

**Authors:** Danon Clemes Cardoso, Maykon Passos Cristiano

**Affiliations:** Departamento de Biodiversidade, Evolução e Meio Ambiente, Universidade Federal de Ouro Preto, Ouro Preto 35400-000, Brazil; maykon@ufop.edu.br

**Keywords:** chromosome evolution, cell biology, genome size, karyotype, ants, genome evolution

## Abstract

**Simple Summary:**

Ants are an important insect group that includes a considerable number of species. Along with this diversity in species, they also exhibit a wide variation in chromosome numbers, from 1 up to 60 chromosomes. DNA molecules can be counted in a specific stage of the cell life cycle and quantified. These DNA molecules are very tightly packed together with several proteins and are called chromosomes. Each species shows a specific number of chromosomes with different shapes and sizes, as well as different quantities of DNA. We can use such information (the number of chromosomes, shape of the chromosomes, and quantity of DNA) as morphological attributes to study evolution at the species level. In this study, we describe new karyotypes of several ant species. In addition, from previous studies, we have compiled all the available information regarding the chromosome number and DNA quantity in fungus-farming ant cells. Different processes, called rearrangements, can change chromosomes over time, producing new character states. Such states can be tracked, along with the species and groups of similar species, using their relationships to identify patterns. We use DNA sequences to reconstruct the relationships of fungus-farming ant species (molecular phylogeny). By comparing such phylogeny with the chromosome number and DNA quantity, we discuss the evolution of chromosomes and DNA quantity (or genome size), and the potential limits to these features across fungus-farming ants.

**Abstract:**

Ants are an important insect group that exhibits considerable diversity in chromosome numbers. Some species show only one chromosome, as in the males of the Australian bulldog ant *Myrmecia croslandi*, while some have as many as 60 chromosomes, as in the males of the giant Neotropical ant *Dinoponera lucida*. Fungus-growing ants are a diverse group in the Neotropical ant fauna, engaged in a symbiotic relationship with a basidiomycete fungus, and are widely distributed from Nearctic to Neotropical regions. Despite their importance, new chromosome counts are scarcely reported, and the marked variation in chromosome number across species has been poorly studied under phylogenetic and genome evolutionary contexts. Here, we present the results of the cytogenetic examination of fungus-farming ants and compile the cytogenetic characteristics and genome size of the species studied to date to draw insights regarding the evolutionary paths of karyotype changes and diversity. These data are coupled with a fossil-calibrated phylogenetic tree to discuss the mode and tempo of chromosomal shifting, considering whether there is an upper limit for chromosome number and genome size in ants, using fungus-farming ants as a model study. We recognize that karyotypes are generally quite variable across fungus-farming ant phylogeny, mostly between genera, and are more numerically conservative within genera. A low chromosome number, between 10 and 12 chromosomes, seems to present a notable long-term evolutionary stasis (intermediate evolutionary stasis) in fungus-farming ants. All the genome size values were inside a limited spectrum below 1 pg. Eventual departures in genome size occurred with regard to the mean of 0.38 pg, indicating that there is a genome, and likely a chromosome, number upper limit.

## 1. Introduction

The genome of any organism is organized into chromosomes that can vary in size, morphology, and DNA composition. Changes in the structure of the chromosomes via mutations and rearrangements, or via numerical changes (aneuploidy) due to errors in the spindle during cell division, can contribute to speciation by impairing gene flow and decreasing fitness [1]. Although there is ample evidence that chromosome changes can contribute to lineage diversification (see [2]), questions and debates remain about the circumstances. However, as morphological traits, chromosomal features are a reliable criterion in evolutionary and taxonomic studies. Among insects, ants display a tremendous diversity of chromosome numbers, varying from *n* = 1 to *n* = 60 (reviewed in [2,3]). This large range in chromosome number attracted the attention of geneticists and raised numerous questions concerning the mechanisms of chromosome evolution in Formicidae. Different authors have described several evolutionary pathways that could better explain the chromosome changes in ants (see [4,5]). Despite the diversity, no more than five percent of the taxonomically described ant species has been studied cytogenetically. The subfamily Myrmicinae contains the highest number of ant karyotypes known to date (see [3]), where the tribe Attini, sub-tribe Attina (fungus-farming ants), covers the widest spectra of the phylogeny. The known fungus-farming ant chromosome numbers range from *n* = 4 to *n* = 27 [3]. Due to advances in microscopy and molecular methodology in the last century, the cytogenetic studies in Formicidae have flourished resulting in more detailed descriptions using sophisticated banding methods and combining molecular phylogenies [6]. Previous studies that have evaluated fungus-farming ants cytogenetically were limited to chromosome number determination, and sometimes, due to the peculiarities of sampling, the analysis of a few individual species, not allowing for the evaluation of the relationships among species within a genus or higher-level taxa [7,8].

Detailed karyotype descriptions (which include the chromosome morphology, size, number, and banding patterns), chromosome counts, and their comparative analyses are important independent tools for use in taxonomy and in understanding chromosome evolution, particularly when relying on the phylogenetic tree [9]. The advances in molecular phylogenetic analysis and the availability of DNA data in public platforms, such as GenBank, have greatly opened up possibilities in the study of chromosomal evolution by inferring evolutionary pathways through combining phylogeny and cytogenetic data in an integrative framework [10,11]. Sequences of protein-coding nuclear genes are useful for resolving phylogenetic relationships within genera and between related species in fungus-growing ants [12], and have been corroborated by inferences based on genomic data [13]. Analysis based on multi-locus data led to a recent molecular phylogenetic hypothesis that resulted in taxonomic changes at the genus level [14,15].

Beyond the number of DNA molecules, estimating the genome content of cell nuclei has contributed to our understanding of genomes through the tree of life. Recently, the nuclear content of approximately 100 ant species was estimated using flow cytometry [16], and now more genome size (GS) ant data has become available. This independent and primary genomic information has provided noteworthy data that can be coupled with cytogenetic data to shed light on the general patterns and processes of the chromosomal changes in fungus-farming ants. Further, cytogenetic and flow cytometry integrated into time-calibrated phylogeny can provide accurate information about the mode and tempo of karyotype evolution. Cytogenetic information, coupled with molecular data, allowed for recent revisionary studies, the identification of sibling species [17], and even the description of a new genus [15].

In this study, we first describe five new karyotypes of fungus-farming ants: *Apterostigma madidiense* (Weber, 1938), *Mycocepurus goeldii* (Forel, 1893), *Sericomyrmex parvulus* (Forel, 1912), *Cyphomyrmex transversus* (Emery, 1894), and *Myrmicocrypta* sp., based on karyomorphometric analysis. We added these karyotypes to the available data on fungus-farming ants in the Ant Chromosome Database (www.ants.ufop.br, accessed on 30 November 2021), depicting the heterochromatic banding pattern and chromosome morphology. We further compiled the genome size information available for the species. All these data were scattered through a time-calibrated phylogeny to evaluate the mode and tempo of chromosomal evolution in fungus-farming ants. We evaluate and discuss our results with regard to chromosome number, morphology, and genome size variation under a phylogenetic framework with a focus on the question: Is there an upper limit of chromosome number for fungus-farming ants? Our intention was not to determine a specific upper number, but to discuss and propose an indication for a limit, considering the GS estimates of ants and the outcomes of the minimum interaction theory. We further discuss the importance of cytogenetic studies.

## 2. Materials and Methods

### 2.1. Colony Sampling

Colonies were sampled from distinct Brazilian states: *A. madidiense* from Cachoeira do Campo, Minas Gerais (MG); *S. parvulus* from Marliéria, Minas Gerais (MG) (S 19°43′21″, W 42°43′26″); *C. transversus* and *Myrmicocripta* sp. from Cabo Frio, Rio de Janeiro (RJ) (S 22°54′29.8″, W 42°02′14.7″); and *M. goeldii* from Morro dos Conventos, Araranguá–Santa Catarina (SC) (S 28°56’08.2″, W 49°21’28″). The sample collection was authorized by the ICMBio (the Chico Mendes Institute for Biodiversity Conservation: special permit 60019). All colonies were excavated and transferred to the Laboratório de Genética Evolutiva e de Populações of the Universidade Federal de Ouro Preto–MG and kept according to the protocols described by Cardoso et al. [18]. The maintenance of the colonies was required to obtain the broods used in the cytogenetic analysis.

### 2.2. Chromosome Preparation and Karyomorphometry

The chromosome slides were obtained from 20 individuals following the protocol described by Imai et al. [4], with small modifications concerning the incubation time, as described by Cardoso et al. [19]. Only high-grade metaphase slides were submitted to conventional staining with 4% Giemsa diluted in Sørensen’s buffer solution at pH 6.8. At least 10 metaphases were photographed using an Olympus BX51 microscope equipped with a digital camera (Olympus DP73). These metaphase chromosomes were evaluated to determine the chromosome number and morphology, as well the karyomorphometric data. Based on the centromere position, the morphology of the chromosomes was sorted following the nomenclature established by Levan et al. [20] as follows: telocentric, subtelocentric, submetacentric, and metacentric, the acronyms for which are t, st, sm, and m, respectively. The karyomorphometric data was obtained by taking measurements from 10 spread metaphases with a clear centromere, chromosomal integrity, and without any overlapping, following the standardized protocol described by Cristiano et al. [21]. The chromosomal features evaluated included the entire length of each chromosome (TL), the length of the long arm of each chromosome (L), the length of the short arm of each chromosome (S), the ratio between the long and short arms (*r* = L/S), and the proportional contribution of each chromosome length (RL) in relation to the total length of all chromosomes (TL × 100/∑TL). The distribution pattern of heterochromatin was obtained using the BSG (barium hydroxide/saline/Giemsa) banding technique following the method described by Sumner [22], with modifications for the duration of treatment with Ba(OH)_2_.

### 2.3. Cytogenetic Data Compilation and Phylogenetic Analysis 

The Ant Chromosome Database (ACdb) (www.ants.ufop.br, accessed on 30 November 2021 [3]) compiles all the information regarding ant chromosome counts and karyotypes published over time, and is regularly updated. We retrieved all fungus-farming ant chromosome counts assembled in the database using the following cytogenetic parameters: diploid chromosome number (2*n*), haploid chromosome number (*n*), and karyotype or karyotypic formulae. The karyotype provides the number of each type of chromosome, whether a chromosome is m, sm, st, t, or acrocentric (a). All the data, including the new counts and karyotypes described here, are presented in Table 1, together with the primary source (author reference). We further extracted the heterochromatic banding pattern of the karyotypes, when available, coding the presence of positive heterochromatin blocks (+) or negative blocks (–) at centromeric (C), pericentromeric (PC), or interstitial (IN) positions, or along the long arm (LA) and/or short arm (SA). We further gathered the genome size information available for fungus-farming ants in Moura et al. [16].

To put the cytogenetic data in a phylogenetic context focusing on fungus-farming genera, coupling the phylogenetic relationship of the species with the karyotype information, we retrieved sequences of five nuclear genes (*elongation factor 1-alpha-F1*, *elongation factor 1-alpha-F2*, *wingless*, *long-wavelength rhodopsin,* and *topoisomerase 1*) from GenBank, which comprised the dataset assembled by Cristiano et al. [15]. We then inferred the calibrated phylogeny using BEAST v2.5 [23] under the fossilized birth–death (FBD) [24] model, setting an uncorrelated, log-normal relaxed clock model to describe the branch-specific substitution rates. The calibration of the parameters and fossils is described by Micolino et al. [17]. Substitution nucleotide models of molecular evolution were estimated for each partition in PartitionFinder 2 [25] (Table S1) and used in the analyses. Independent MCMC analyses were run for 100 million generations, sampling every 1000 generations. Runs were evaluated using Tracer v1.7 [26] with effective sample size (ESS) values for all parameters over 200. The first 20% of the sampled tree topologies were discarded as burn-in, and the remaining trees were summarized using Treeannotator v2.5, after the removal of all fossils using the FullToExtantTreeConverter tool implemented in Beauti v2.5 [23]. The Figtree software [27] was utilized to visualize the final tree with credible intervals and branch labels (Figure S1).

## 3. Results 

**Figure 1:**
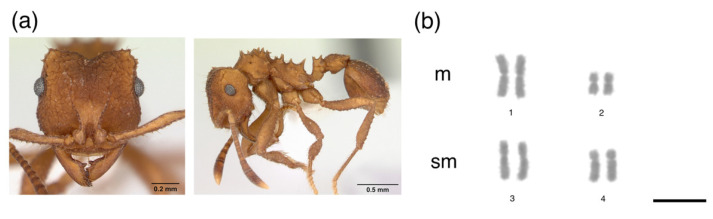
Mitotic chromosomes of *Mycocepurus goeldii* stained with Giemsa. Species (**a**) images and (**b**) diploid karyotype of *Mycocepurus goeldii* with 2*n* = 8 chromosomes. m = metacentric, sm = submetacentric. Scale bar = 5 µm. Ant image from AntWeb (www.antweb.org, accessed on 13 June 2021): *Mycocepurus goeldii* (CASENT0173988, photo by: A. Nobile).

**Figure 2:**
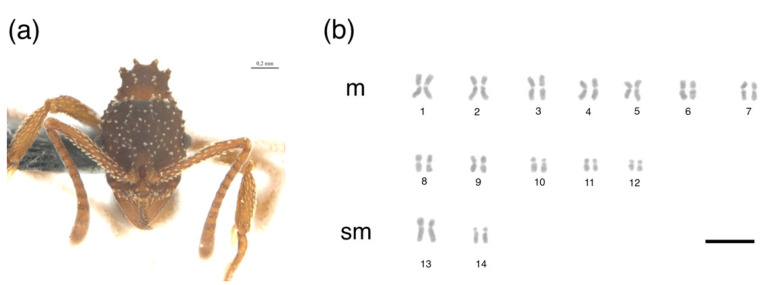
Mitotic chromosomes of *Myrmicocripta* sp. stained with Giemsa. Species (**a**) images and (**b**) diploid karyotype of *Myrmicocripta* sp. with 2*n* = 28 chromosomes. m = metacentric; sm = submetacentric. Scale bar = 5 µm. Ant image was obtained in our lab.

**Figure 3:**
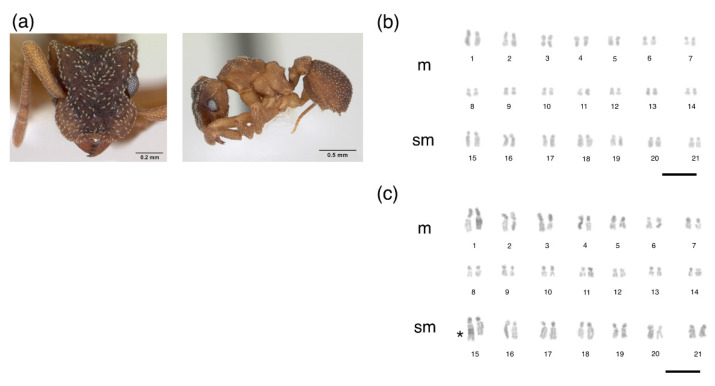
Mitotic chromosomes of *Cyphomyrmex transversus* stained with Giemsa. Species (**a**) images and (**b**,**c**) diploid karyotype of *Cyphomyrmex transversus* with 2*n* = 42 chromosomes. m = metacentric, sm = submetacentric. Polymorphism in chromosome pair 15 is marked by an asterisk. Scale bar = 5 µm. Ant image from AntWeb (www.antweb.org, accessed on 13 June 2021): *Cyphomyrmex transversus* (CASENT0173958, photo by: A. Nobile).

**Figure 4:**
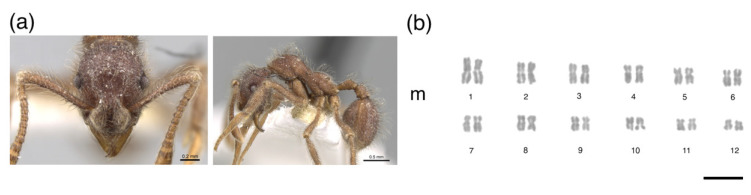
Mitotic chromosomes of *Apterostigma madidiense* stained with Giemsa. Species (**a**) images and (**b**) diploid karyotype of *Apterostigma madidiense* with 2*n* = 24 chromosomes. m = metacentric. Scale bar = 5 µm. Ant image from AntWeb (www.antweb.org, accessed on 13 June 2021): *Apterostigma madidiense* (CASENT0281778, photo by: S. Hartman).

**Figure 5:**
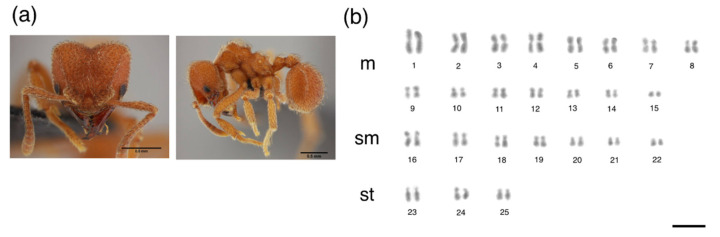
Mitotic chromosomes of *Sericomyrmex parvulus* stained with Giemsa. Species (**a**) images and (**b**) diploid karyotype of *Sericomyrmex parvulus* with 2*n* = 50 chromosomes. m = metacentric; sm = submetacentric; st = subtelocentric. Scale bar = 5 µm. Ant image from AntWeb (www.antweb.org, accessed on 13 June 2021): *Sericomyrmex parvulus* (UFV-LABECOL-000372, photo by: J. Chaul).

The metaphases of *M. goeldii* consisted of eight chromosomes in a diploid set (2*n* = 8, Figure 1), 2 K = 6 m + 2 sm and the fundamental number (FN) = 16 (Table 1). The metaphases of *Myrmicocripta* sp. consisted of 28 chromosomes in the diploid set (2*n* = 28, Figure 2), 2 K = 24 m + 4 sm and FN = 56 (Table 2). The metaphases of *C. transversus* consisted of 42 chromosomes in the diploid set (2*n* = 42, Figure 3), 2 K = 28 m + 14 sm and FN = 84 (Table 3). Some individuals from the same colony bore a polymorphism in submetacentric pair 15 (Figure 4). The metaphases of *A. madidiense* consisted of 24 chromosomes in the diploid set (2*n* = 24, Figure 4), 2 K = 24 m and FN = 48 (Table 4). The metaphases of *S. parvulus* consisted of 2*n* = 50 (Figure 5), 2 K = 30 m + 14 sm + 6 st and FN = 100. We were not able to establish detailed karyomorphometric data for *S. parvulus* due to the limited number of high-quality spreads (below 10). The heterochromatic pattern of the species studied here are summarized in Table 5.

**Figure 6:**
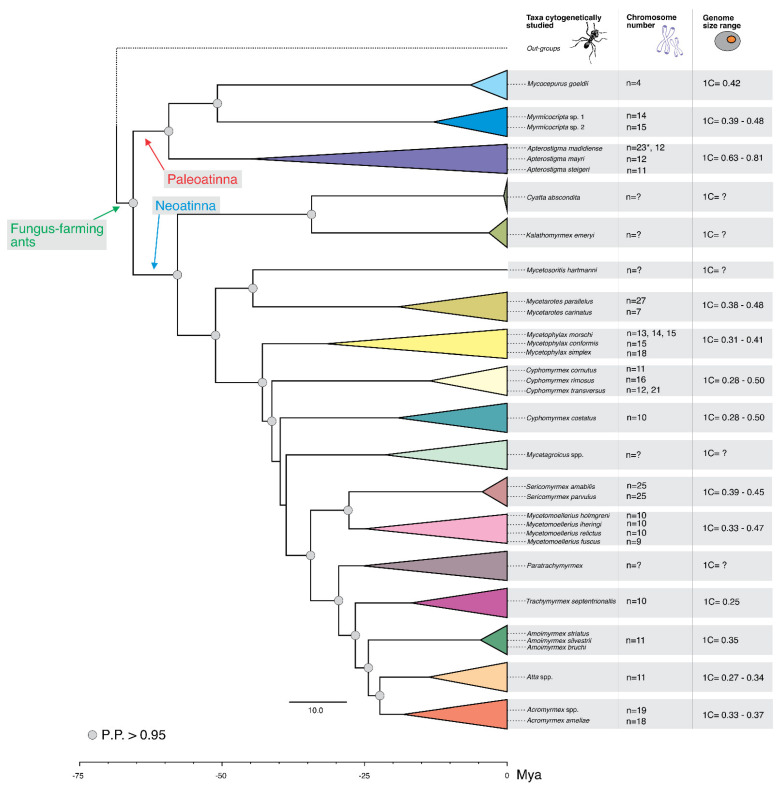
Cytogenetic information regarding fungus-farming ants mapped on the time-calibrated phylogenetic tree of subtribe Attina (Myrmicinae) aiming genera. Available chromosome numbers and genome size for certain taxa are given (data refer to Table 5). Asterisk: previously published chromosome report that differs from the number published in the present study.

Taking into account the new information established here and the available cytogenetic data, there are 58 chromosome counts of fungus-farming ants (Table 5). From these, 10 are chromosome counts recorded for specimens not identified at the species level, whereas seven counts are related to the same taxa with distinct karyotypes from different populations. Nevertheless, genome size estimates are available for 31 species. The estimated GSs are all around the mean genome size of 0.38 pg, with two exceptions: *Apterostigma* spp. (0.61–0.81 pg) and *C. transversus* (0.50 pg). Spreading the cytogenetic and GS data across the calibrated phylogeny that was generated, we detected wide chromosomal and genome size variability among the species of fungus-farming ants (Figure 6). Although limited to the number of observations, there is a clear pattern where karyotypes with a haploid number of 10 to 12 are distributed along the phylogeny, and most species bear a GS of below 0.40 pg. Higher chromosome numbers (*n* > 15) are restricted to some lineages within the genera. Nevertheless, even higher chromosome numbers (*n* > 25) are rare and restricted to lineages of Neoattini (Figure 6). Our data allow for the identification of a general pattern indicating lineages that are recently divergent, splitting below 26 Mya, and that are more homogenous with regard to the number of chromosomes (Figure 6). For instance, the known *Trachymyrmex* species show a haploid set of 10 chromosomes, and all *Amoimyrmex* and *Atta* show a haploid set of 11 chromosomes (Figure 6). The compiled heterochromatic pattern data suggest that karyotypes under 15 haploid chromosomes show heterochromatin restricted to the centromere, whereas karyotypes with a higher number of chromosomes (*n* > 15) show a higher number of positive heterochromatic blocks, including chromosomes with a heterochromatic long arm (Table 5).

## 4. Discussion

### 4.1. Chromosome Number, Mode, and Tempo of Karyotype Change 

Genes are scattered across the genome, which in eukaryotes, are organized and distributed in a different number of DNA molecules with differing extents. These molecules are complexed with proteins and comprise the chromosomes that are ultimately the units of inheritance. Ants exhibit an astonishing variation in chromosome number and structure (e.g., [17]) that, it has been postulated, take part in ant speciation and diversification. They show a haplodiploid sex-determination system, as in all Hymenoptera, with haploid chromosome numbers ranging from *n* = 1 to *n* = 60, and include diverse karyotypes comprising various types of chromosomes (see [2]. Of the subfamilies of Formicidae, Myrmicinae is the subfamily with the greatest number of studied species and taxa from a cytogenetic perspective [3]. Here, we have added six new chromosome counts to the remarkable fungus-farming ants that live in symbiosis with Basidiomycete fungi inside their subterranean nests across the Americas.

According to our results, approximately 58 taxa in 13 out of 18 genera have been studied. Considering only the published data, the diploid karyotype of fungus-farming ants varies from 4 haploid chromosomes in *Mycocepurus goeldii* (Forel, 1893) up to 27 haploid chromosomes in *Mycetarotes parallelus* (Roger, 1863). The diploid chromosome numbers are, in general, variable in fungus-farming ants, as is the genome size (Figure 6). However, the variability in chromosome number and genome size is highly unequally distributed among fungus-farming ant genera, with most of the variability concentrated in the early diverged lineages/genera, such as in *Mycetophylax* and *Mycetarotes*. In contrast, other lineages have much more stable chromosome numbers, such as *n* = 11 in the leafcutting ants from the genera *Atta* and *Amoimyrmex*, as well as *n* = 19 in the leafcutting ants from the genus *Acromyrmex.*

The chromosome number *n* = 10 or *n* = 11 exhibits a notable long-term evolutionary stasis across fungus-farming ant lineages (see Figure 6), also refereed as intermediate evolutionary statis. Karyotypes with *n* = 11 chromosomes were identified in the *Apterostigma* clade and in the *Cyphomyrmex* clade dated to ~45 Mya and ~25 Mya, respectively. The same chromosome number was identified in *Amoimyrmex* and *Atta* clades that are much more recent, estimated to ~4.7 Mya and ~13.7 Mya, respectively. However, the *n* = 10 chromosomes that were identified in the *Cyphomyrmex* clade (“costatus group”), the *Mycetomollerius* clade, and the *Trachymyrmex* clade are estimated at approximately ~19 Mya, ~24.8 Mya, and ~16.8 Mya, respectively (Figure 6 and Figure S1). This pattern of chromosome number variation and distribution was also observed in other Hymenoptera groups, such as stingless bees [46] and parasitoid wasps [47].

Considering the phylogenetic relationship of fungus-farming species, the general slight variation in genome size across lineages, with few exceptions, shows that a low chromosome number between 10 to 12 haploid chromosome and genome size near to 0.38 pg (see Figure 6), should be plesiomorphic features. Indeed, a low ancestral GS of ~0.37–0.38 has been estimated and proposed for Formicidae [16] and the chromosome number between 10 to 12 of metacentric/submetacentric chromosomes spreads widely across fungus-farming ant lineages. Thus, considering the tight GS in fungus-farming ants, the observed differences in chromosome number across fungus-farming ant genera suggests that changes in chromosome numbers evolve gradually over time, via multiple rearrangement events that culminate in different races due to the fixation of a single or a few chromosomal changes, followed by the extinction of intermediate karyotypes (see [10,17,48,49,50]). Therefore, karyotypes *n* ≤ 15 and GSs of approximately 0.38 pg should be frequent in fungus-farming ants, whereas karyotypes *n* > 15 and GS > 0.38 pg are exceptions and are restricted to only some fungus-farming ant lineages. The accumulation of repetitive DNA appears to be mainly responsible for genome size expansions, instead of genome duplication see [16]. These increases in repetitive DNA may promote changes in chromosome number since the heterochromatic regions are hotspots for chromosome breakpoints [51]. In fact, given its nature and critical function, heterochromatin is considered to be a rapidly evolving genomic component that can promote species divergence [52]. As an example, there is a small GS variation among leafcutting ants (*Atta*, *Acromyrmex,* and *Amoimyrmex*), and the chromosome number is indeed consistent within each genus (Figure 6). However, in contrast with *Atta* and *Amoimyrmex*, the karyotypes of *Acromyrmex* are *n* = 19 (*n* > 15) and show a great distribution of heterochromatic regions, as revealed by the C-banding [32]. Similarly, *Mycetophylax simplex n* = 18 (*n* > 15) also shows a much higher number of C-banded heterochromatic regions compared with the sister species studied cytogenetically [10].

Chromosomal rearrangements should thus promote species isolation and lineage diversification because of the reduced recombination between heterokaryotypes [53]. The rate at which chromosomes change may be related to the amount and distribution of heterochromatin. The repetitive DNA comprising heterochromatin may promote rearrangements by relaxing the putative selective pressure on gene synteny and linkage groups. Indeed, the poorly heterochromatin-rich karyotypes show a chromosome number of *n* ≤ 15, as observed in *Mycetomollerius* and *Cyphomyrmex* (see Table 1 and Figure 6). Most fungus-farming ants studied to date show heterochromatin restricted to the centromeric region [7,10,11,32,37,43]. Further studies covering more data and combining genomic information may shed more light on the above-proposed hypothesis of chromosome number variation and diversity.

Karyotype evolution in ants is commonly explained in terms of the “minimum interaction theory—MIT” proposed by Imai et al. [4,5] as a general mechanism to explain the chromosomal change in ants. Following the MIT, over evolutionary time, the number of chromosomes in the ant karyotypes will increase, whereas chromosome size will decrease. Such a pattern minimizes the threat of deleterious rearrangements due to the interaction of chromosomes inside the nucleus. The predicted increase in chromosome number is due to centric fission that represents the main chromosome rearrangements in ants, followed by chromatin addition (mainly heterochromatin) or pericentromeric inversions (see [5]). Although the minimum interaction theory does not disregard fusions, such a rearrangement is considered rare and fixed or positively selected when it brings about short-term advantages [5,48].

Accordingly, in the course of evolution, chromosomes will increase in number and reduce in size. Taking the cytogenetic data available to date, phylogenetic relationships, and the time of divergence studied here, a higher chromosome number would be expected in ancient lineages than in recent lineages. The higher chromosome number known is observed in *Mycetarotes parallelus* (*n* = 27), but unexpectedly low karyotypes, such as *n* < 15, are found in the anciently divergent clade called Paleoattina, which comprises *Mycocepurus*, *Myrmicocripta,* and *Aptersotigma* (Figure 6). Furthermore, higher counts, such as *n* = 25 or *n* = 19 (*n* > 15), are scattered in the clade comprising recently divergent lineages such as *Sericomyrmex* and *Acromyrmex*. The cytogenetic data plotted in the phylogeny reveals that the karyotype *n* = 10, or a number close to that (*n* = 11 and 12), is the most common karyotype across fungus-farming ant genera, showing that the karyotype of the lineages is around the average or the most frequent number. Thus, we do not discard that the minimum interaction theory may be a driving force behind karyotype evolution in ants; however, it seems that other forces may regulate chromosome number by imposing a general common karyotype number, such as *n* < 15, in fungus-farming ants. A few exceptions where the karyotype reaches higher chromosome numbers appear to overcome the barrier likely caused by the expansion of repetitive genomic elements [16]. This is in agreement with the heterochromatic pattern observed in fungus-farming ants. Karyotypes with a haploid number over 15 chromosomes show a higher number of heterochromatic-positive bands. This also matches with the MIT, which presumes that after centric fission occurs, the duplication of repetitive regions at the extreme of the newly produced telocentric/acrocentric chromosomes creates stabilization [5]. Such processes will increase the number of heterochromatic segments and will promote the increase in GS, not as abruptly as polyploidization, but as we observed across the estimated GS values [16,54] (see Figure 6).

### 4.2. Is There an Upper Limit to the Chromosome Number of Fungus-Farming Ants?

The answer to that question is intriguing and complex. The chromosome number ultimately means how many DNA molecules the genome of any organism comprises. A fundamental property of the genome is how the genes are distributed along and among the chromosomes, which has a strong influence on gene function and regulation. Ants exhibit an astonishing variation in chromosome numbers, from a species that starts with a minimum of *n* = 1 in the Australian bulldog ant *Myrmecia croslandi* [55], only possible due to the haplodiploid sex determination, up to as much as *n* = 60 in the giant neotropical ant *Dinoponera lucida* [56]. In contrast, such a huge diversity is not observed across known genome size values, which vary from 0.18 to 0.81 pg [16]. Chromosome numbers vary on a scale of 60 times (from 1 to 60), whereas genome size is below 4.5 times. Presumably, there is an evolutionary constraint preventing genome expansion up to a certain limit, and similarly, there would be an upper limit to chromosome number. Thus, the answer to our initial question is: yes!

The maximum number of chromosomes known thus far for fungus-farming ants is *n* = 27 for one record and *n* = 25 for another (see Table 1). The scarcity of karyotypes with large chromosome numbers and the high frequency of karyotypes with *n* < 15 suggests a potential constraint in chromosome number. Here, based on genome size data in which great departures from the mean GS are rare, we propose that there is an upper limit for chromosome number. If not, we would observe karyotypes above *n* = 15 more often and higher GS estimates. According to the MIT, the higher the chromosome number is, the smaller the chromosomes are, and this is a fact observed in our karyomorphometric data. In addition, according to the MIT, centric fission events producing unstable telocentric/acrocentric chromosomes promote heterochromatin expansion, resulting in an increase in DNA [5]. However, the limited range of GSs observed in fungus-farming ants is the first instance of evidence for an upper limit in chromosome numbers. Fusion events counterbalance centric fissions due to the loss of DNA see [4,5]. Furthermore, the progressive reduction in the size of the chromosomes as stated by the MIT may increase the risk of mis-segregation of chromosomes during cell division and dysploidy [57]. For instance, the species *Dinoponera lucida*, which bears the highest number of chromosomes in Formicidae, shows huge variation in chromosome number across populations [56].

Several hypotheses have been invoked and tested to explain the tight genome size of ants: the developmental strategy (holometabolous) and metabolic rate have been suggested as constraints against genome size expansion, as well as the eusociality [58,59]. Similarly, such mechanisms may regulate the upper limit of chromosomes in ants. A GS above 0.70 pg is rare, and is suggested to eventually occur from whole-genome duplication (double the mean GS of 0.38 pg). Most ants show a GS of between 0.25 and 0.50 pg, and such a range is suggested to be the result of transposition element (TE) activity and repetitive DNA expansion rather than whole-genome duplication [16]. It is a fact that the ant genome sizes estimated so far are always below 1 pg. There are still open questions that need to be addressed, including: which genetic mechanism is triggered? And which evolutionary process may cause the loss and gain of DNA in ants maintaining such a small genome? Some evidence suggests that DNA loss is likely to be favored compared with gain by selection to minimize genetic instability, or phenotypic effects due to the required changes in the nucleus and cell size [60]. Furthermore, there are biochemical and energy costs associated with maintaining expanded genomes, as well the cellular machinery involved in chromosome organization and splitting during cell division [61]. This is in agreement with the minimum interaction theory and the implicated processes (centric fissions) responsible for the chromosomal change (see [5]); however, we hypothesize that there is a balance between fission and fusion events. The constricted genome size, the expansion constraints, the TE activity promoting insertions, and duplication may trigger chromosome rearrangements within an upper limit number of DNA molecules. Thus, there is an upper limit in chromosome number, but not for karyotype shifting (inversions, translocations, etc.), which may explain the dynamic and diverse karyotype of ants. Further genomic data and chromosome-level assembly studies may shed light on ant chromosome biology. It is a fact that there are, in nature, karyotypes bearing over hundreds of chromosomes, such as in the butterfly *Polyommatus* (*Plebicula*) *atlanticus* (*n* = circa 223) [62]. However, it seems they are the exceptions, rather than the rule.

### 4.3. Chromosome Counts: How Far We Get and What Is Still Needed

In this paper, we evaluated the currently available cytogenetic information on fungus-farming ants, a distinguishable group of neotropical fauna. From the data studied to date, it is possible to make some assumptions regarding chromosome numbers, karyotype evolution, and genome size variation. Among all the ant species, the highest amount of cytogenetic data available is on the Myrmicinae, specifically fungus-farming ants. However, considering the diversity of ants and their ecological importance, we have only superficially touched on a much deeper compendium of knowledge when we consider that less than five percent of ants has been studied cytogenetically thus far [3]. In our opinion, the chromosome number is still essential, and we need to continue counting. Furthermore, we need to couple cytogenetic information with molecular and genomic data. Considering the generally tight genome size of ants and the karyotype diversity, the interplay between fission–fusion and other structural rearrangements, such as duplication and inversions, may govern karyotype evolution in fungus-farming ants. We can also extrapolate this to all ants. It is likely that changes in chromosome number caused by fission, fusion, inversions, and duplications may have contributed to ant diversification [2,3]. Thus, cytogenetic studies can definitely contribute to ant systematics, taxonomy, and evolutionary biology.

## 5. Conclusions

In this paper, we reported two chromosome counts that differ from previously published chromosome reports for the same species. This raises issues that need to be addressed in an effort to gain cytogenetic knowledge and application: first, an unidentified species may be tagged with cytochrome oxidase I (*cox 1* or COI) sequences for further identification (see [63]); second, different counts may suggest independent lineages and new potential species; third, chromosome counting needs protocol scrutiny with regard to the number of cell/metaphases assessed in colonies/individuals to avoid flawed reports. Bearing in mind the well-known case of the human karyotype where, for more than 30 years, it was thought to be 48 diploid chromosomes instead of 46 (see [64]), in the chromosome counting of ants, and Hymenoptera in general, evaluating a minimum of individuals and cells should be considered. Other issues that require attention are the observance of source tissue (male or female), the number of cells/individuals per colony, and the number of colonies. The haplodiploid sex determination prevents chromosome paring from male samples, which could result in a misinterpretation of numbers. Increasing the number of cells/individuals/colonies decreases the propensity of errors. Due to sampling difficulties or bias, highlighting the concerns in reporting such data is necessary for future studies. In conclusion, the phylogenetic relationship of fungus-farming ants and the cytogenetic data presented here suggest that a low chromosome number between 10 to 12 haploid chromosome and genome size near to 0.38 pg should be plesiomorphic features. The karyotype variation observed today is a result of an interplay among distinct chromosomal rearrangements and likely lineage-specific.

## Figures and Tables

**Table 1:** Karyomorphometric analyses of the chromosomes of *Mycocepurus goeldii*. All measurements are given in “μm”. TL: total length; L: long arm length; S: short arm length; RL: relative length; r: arm ratio; KL: karyotype length.

Chromosome (Homologue)	TL (±SD)	L (±SD)	S (±SD)	RL (±SD)	r (±SD)	Classification
1	8.79 ± 3.04	4.78 ± 1.71	4.01 ± 1.37	15.70 ± 0.35	1.19 ± 0.13	Metacentric
(1)	8.37 ± 3.03	4.48 ± 1.6	3.89 ± 1.46	14.89 ± 0.64	1.16 ± 0.13	Metacentric
2	4.82 ± 1.57	2.82 ± 1.00	1.99 ± 0.57	08.66 ± 0.37	1.40 ± 0.13	Metacentric
(2)	4.64 ± 1.52	2.64 ± 0.89	2.00 ± 0.64	08.34 ± 0.35	1.32 ± 0.12	Metacentric
3	8.02 ± 3.03	4.97 ± 1.73	3.05 ± 1.38	14.19 ± 0.87	1.70 ± 0.28	Submetacentric
(3)	7.76 ± 2.98	4.87 ± 1.97	2.99 ± 1.03	13.70 ± 0.91	1.61 ± 0.23	Submetacentric
4	6.90 ± 2.16	4.90 ± 1.62	2.00 ± 0.58	12.47 ± 1.04	2.45 ± 0.36	Submetacentric
(4)	6.67 ± 2.14	4.65 ± 1.50	2.03 ± 0.66	12.05 ± 0.90	2.29 ± 0.20	Submetacentric
KL	55.97					

**Table 2:** Karyomorphometric analyses of the chromosomes of *Myrmicocripta* sp. All measurements are given in “μm”. TL: total length; L: long arm length; S: short arm length; RL: relative length; r: arm ratio; KL: karyotype length.

Chromosome (Homologue)	TL (±SD)	L(±SD)	S(±SD)	RL(±SD)	r(±SD)	Classification
1	4.27 ± 0.88	2.37 ± 0.44	1.89 ± 0.46	5.20 ± 0.18	1.28 ± 0.15	Metacentric
(1)	4.14 ± 0.89	2.27 ± 0.44	1.87 ± 0.46	5.03 ± 0.15	1.23 ± 0.13	Metacentric
2	3.96 ± 0.83	2.24 ± 0.49	1.72 ± 0.38	4.82 ± 0.18	1.32 ± 0.16	Metacentric
(2)	3.89 ± 0.81	2.12 ± 0.46	1.77 ± 0.37	4.73 ± 0.17	1.21 ± 0.10	Metacentric
3	3.76 ± 0.89	2.07 ± 0.57	1.69 ± 0.38	4.54 ± 0.20	1.23 ± 0.22	Metacentric
(3)	3.68 ± 0.89	1.99 ± 0.49	1.69 ± 0.43	4.44 ± 0.18	1.19 ± 0.14	Metacentric
4	3.44 ± 0.79	1.95 ± 0.50	1.49 ± 0.31	4.16 ± 0.14	1.30 ± 0.13	Metacentric
(4)	3.30 ± 0.75	1.83 ± 0.39	1.47 ± 0.37	4.00 ± 0.12	1.26 ± 0.10	Metacentric
5	3.22 ± 0.71	1.79 ± 0.38	1.43 ± 0.34	3.91 ± 0.11	1.26 ± 0.11	Metacentric
(5)	3.14 ± 0.71	1.70 ± 0.34	1.43 ± 0.37	3.80 ± 0.09	1.21 ± 0.11	Metacentric
6	3.06 ± 0.69	1.69 ± 0.37	1.37 ± 0.34	3.70 ± 0.10	1.25 ± 0.15	Metacentric
(6)	2.99 ± 0.65	1.68 ± 0.37	1.31 ± 0.31	3.63 ± 0.06	1.29 ± 0.13	Metacentric
7	2.90 ± 0.63	1.62 ± 0.35	1.28 ± 0.31	3.51 ± 0.08	1.28 ± 0.14	Metacentric
(7)	2.86 ± 0.64	1.57 ± 0.31	1.29 ± 0.33	3.46 ± 0.07	1.24 ± 0.12	Metacentric
8	2.80 ± 0.64	1.57 ± 0.35	1.24 ± 0.31	3.39 ± 0.06	1.29 ± 0.14	Metacentric
(8)	2.73 ± 0.62	1.54 ± 0.34	1.20 ± 0.29	3.31 ± 0.10	1.30 ± 0.14	Metacentric
9	2.65 ± 0.63	1.47 ± 0.31	1.17 ± 0.32	3.20 ± 0.14	1.28 ± 0.16	Metacentric
(9)	2.56 ± 0.63	1.41 ± 0.33	1.15 ± 0.31	3.09 ± 0.16	1.25 ± 0.17	Metacentric
10	2.41 ± 0.57	1.39 ± 0.29	1.01 ± 0.30	2.91 ± 0.17	1.42 ± 0.20	Metacentric
(10)	2.20 ± 0.55	1.22 ± 0.31	0.97 ± 0.30	2.65 ± 0.20	1.37 ± 0.16	Metacentric
11	1.94 ± 0.48	1.11 ± 0.27	0.82 ± 0.25	2.33 ± 0.11	1.43 ± 0.17	Metacentric
(11)	1.84 ± 0.44	1.06 ± 0.26	0.79 ± 0.20	2.22 ± 0.10	1.36 ± 0.17	Metacentric
12	1.70 ± 0.39	0.98 ± 0.22	0.72 ± 0.18	2.06 ± 0.11	1.38 ± 0.13	Metacentric
(12)	1.62 ± 0.38	0.93 ± 0.21	0.69 ± 0.17	1.95 ± 0.09	1.35 ± 0.13	Metacentric
13	3.57 ± 0.82	2.39 ± 0.53	1.17 ± 0.31	4.34 ± 0.46	2.07 ± 0.26	Submetacentric
(13)	3.26 ± 0.85	2.19 ± 0.53	1.07 ± 0.31	3.99 ± 0.63	2.08 ± 0.11	Submetacentric
14	2.36 ± 0.50	1.67 ± 0.39	0.70 ± 0.12	2.88 ± 0.20	2.38 ± 0.30	Submetacentric
(14)	2.22 ± 0.43	1.52 ± 0.31	0.70 ± 0.15	2.72 ± 0.22	2.18 ± 0.30	Submetacentric
KL	82.47					

**Table 3:** Karyomorphometric analyses of the chromosomes of *Cyphomyrmex transversus*. All measurements are given in “μm”. TL: total length; L: long arm length; S: short arm length; RL: relative length; r: arm ratio; KL: karyotype length.

Chromosome (Homologue)	TL (±SD)	L(±SD)	S(±SD)	RL(±SD)	r(±SD)	Classification
1	3.19 ± 1.40	1.76 ± 0.73	1.43 ± 0.68	3.84 ± 0.2	1.27 ± 0.14	Metacentric
(1)	2.91 ± 1.29	1.71 ± 0.76	1.20 ± 0.55	3.50 ± 0.22	1.45 ± 0.22	Metacentric
2	2.62 ± 1.06	1.40 ± 0.62	1.22 ± 0.47	3.18 ± 0.18	1.18 ± 0.12	Metacentric
(2)	2.48 ± 1.09	1.31 ± 0.62	1.17 ± 0.50	2.98 ± 0.23	1.28 ± 0.16	Metacentric
3	2.39 ± 1.04	1.28 ± 0.53	1.10 ± 0.52	2.87 ± 0.24	1.22 ± 0.17	Metacentric
(3)	2.23 ± 0.94	1.31 ± 0.53	0.92 ± 0.43	2.69 ± 0.21	1.44 ± 0.22	Metacentric
4	2.13 ± 0.92	1.26 ± 0.59	0.86 ± 0.33	2.56 ± 0.14	1.44 ± 0.20	Metacentric
(4)	2.08 ± 0.89	1.18 ± 0.5	0.90 ± 0.40	2.51 ± 0.10	1.36 ± 0.19	Metacentric
5	2.00 ± 0.84	1.13 ± 0.43	0.88 ± 0.43	2.42 ± 0.09	1.34 ± 0.26	Metacentric
(5)	1.89 ± 0.71	1.10 ± 0.42	0.79 ± 0.30	2.32 ± 0.14	1.40 ± 0.13	Metacentric
6	1.83 ± 0.67	1.03 ± 0.37	0.80 ± 0.33	2.24 ± 0.12	1.32 ± 0.23	Metacentric
(6)	1.80 ± 0.65	0.97 ± 0.31	0.82 ± 0.36	2.20 ± 0.11	1.26 ± 0.13	Metacentric
7	1.75 ± 0.65	0.97 ± 0.37	0.79 ± 0.28	2.15 ± 0.10	1.25 ± 0.16	Metacentric
(7)	1.72 ± 0.63	0.95 ± 0.35	0.77 ± 0.30	2.11 ± 0.08	1.34 ± 0.12	Metacentric
8	1.68 ± 0.63	0.98 ± 0.33	0.72 ± 0.29	2.05 ± 0.11	1.39 ± 0.14	Metacentric
(8)	1.66 ± 0.62	0.94 ± 0.37	0.72 ± 0.27	2.03 ± 0.11	1.36 ± 0.18	Metacentric
9	1.62 ± 0.60	0.95 ± 0.38	0.68 ± 0.26	1.99 ± 0.08	1.42 ± 0.22	Metacentric
(9)	1.59 ± 0.60	0.91 ± 0.37	0.68 ± 0.26	1.95 ± 0.10	1.42 ± 0.18	Metacentric
10	1.55 ± 0.60	0.89 ± 0.36	0.66 ± 0.25	1.89 ± 0.08	1.33 ± 0.13	Metacentric
(10)	1.54 ± 0.60	0.85 ± 0.30	0.69 ± 0.29	1.88 ± 0.07	1.27 ± 0.13	Metacentric
11	1.52 ± 0.56	0.82 ± 0.28	0.70 ± 0.28	1.86 ± 0.08	1.21 ± 0.15	Metacentric
(11)	1.48 ± 0.55	0.83 ± 0.30	0.66 ± 0.25	1.82 ± 0.07	1.26 ± 0.08	Metacentric
12	1.45 ± 0.50	0.85 ± 0.31	0.60 ± 0.20	1.79 ± 0.10	1.43 ± 0.16	Metacentric
(12)	1.41 ± 0.50	0.81 ± 0.28	0.60 ± 0.22	1.74 ± 0.10	1.38 ± 0.14	Metacentric
13	1.38 ± 0.49	0.78 ± 0.26	0.59 ± 0.24	1.69 ± 0.08	1.37 ± 0.21	Metacentric
(13)	1.37 ± 0.49	0.77 ± 0.25	0.59 ± 0.24	1.68 ± 0.08	1.34 ± 0.15	Metacentric
14	1.34 ± 0.49	0.75 ± 0.28	0.59 ± 0.21	1.64 ± 0.09	1.28 ± 0.10	Metacentric
(14)	1.27 ± 0.44	0.76 ± 0.29	0.51 ± 0.16	1.56 ± 0.11	1.46 ± 0.14	Metacentric
15	2.72 ± 1.32	1.90 ± 0.95	0.82 ± 0.38	3.29 ± 0.73	2.28 ± 0.19	Submetacentric
(15)	2.58 ± 1.20	1.80 ± 0.84	0.78 ± 0.37	3.14 ± 0.71	2.33 ± 0.28	Submetacentric
16	2.60 ± 1.00	1.80 ± 0.67	0.80 ± 0.33	3.18 ± 0.15	2.27 ± 0.17	Submetacentric
(16)	2.49 ± 0.93	1.75 ± 0.69	0.74 ± 0.25	3.06 ± 0.14	2.32 ± 0.21	Submetacentric
17	2.43 ± 0.94	1.69 ± 0.64	0.75 ± 0.31	2.97 ± 0.07	2.27 ± 0.18	Submetacentric
(17)	2.34 ± 0.98	1.62 ± 0.65	0.72 ± 0.33	2.84 ± 0.09	2.29 ± 0.15	Submetacentric
18	2.18 ± 0.95	1.48 ± 0.62	0.71 ± 0.33	2.64 ± 0.16	2.11 ± 0.16	Submetacentric
(18)	2.13 ± 0.94	1.47 ± 0.68	0.66 ± 0.26	2.56 ± 0.15	2.21 ± 0.25	Submetacentric
19	2.04 ± 0.93	1.39 ± 0.59	0.65 ± 0.35	2.45 ± 0.17	2.19 ± 0.21	Submetacentric
(19)	2.00 ± 0.90	1.35 ± 0.61	0.66 ± 0.30	2.41 ± 0.18	2.07 ± 0.21	Submetacentric
20	1.91 ± 0.78	1.27 ± 0.50	0.64 ± 0.29	2.32 ± 0.11	2.01 ± 0.16	Submetacentric
(20)	1.82 ± 0.76	1.24 ± 0.54	0.59 ± 0.23	2.21 ± 0.17	2.08 ± 0.26	Submetacentric
21	1.64 ± 0.75	1.10 ± 0.52	0.54 ± 0.23	1.99 ± 0.25	2.02 ± 0.17	Submetacentric
(21)	1.52 ± 0.75	1.03 ± 0.55	0.50 ± 0.20	1.83 ± 0.22	2.00 ± 0.21	Submetacentric
KL	82.28					

**Table 4:** Karyomorphometric analyses of the chromosomes of *Apterostigma madidiense*. All measurements are given in “μm”. TL: total length; L: long arm length; S: short arm length; RL: relative length; r: arm ratio; KL: karyotype length.

Chromosome (Homologue)	TL (±SD)	L(±SD)	S(±SD)	RL(±SD)	r(±SD)	Classification
1	6.95 ± 1.32	3.73 ± 0.81	3.22 ± 0.52	6.74 ± 0.25	1.17 ± 0.09	Metacentric
(1)	6.58 ± 1.21	3.54 ± 0.69	3.06 ± 0.57	6.39 ± 0.24	1.17 ± 0.07	Metacentric
2	5.75 ± 1.12	3.13 ± 0.63	2.62 ± 0.46	5.58 ± 0.23	1.19 ± 0.07	Metacentric
(2)	5.39 ± 1.05	2.92 ± 0.54	2.45 ± 0.52	5.22 ± 0.18	1.20 ± 0.13	Metacentric
3	5.01 ± 0.89	2.77 ± 0.44	2.24 ± 0.48	4.87 ± 0.15	1.26 ± 0.18	Metacentric
(3)	4.82 ± 0.86	2.79 ± 0.49	2.03 ± 0.39	4.68 ± 0.13	1.38 ± 0.18	Metacentric
4	4.71 ± 0.82	2.61 ± 0.43	2.09 ± 0.53	4.58 ± 0.12	1.36 ± 0.16	Metacentric
(4)	4.60 ± 0.79	2.63 ± 0.41	1.97 ± 0.56	4.47 ± 0.09	1.48 ± 0.15	Metacentric
5	4.50 ± 0.74	2.54 ± 0.32	1.94 ± 0.44	4.38 ± 0.08	1.35 ± 0.19	Metacentric
(5)	4.38 ± 0.72	2.54 ± 0.35	1.83 ± 0.41	4.26 ± 0.07	1.42 ± 0.21	Metacentric
6	4.31 ± 0.73	2.56 ± 0.40	1.73 ± 0.36	4.19 ± 0.07	1.50 ± 0.17	Metacentric
(6)	4.25 ± 0.71	2.36 ± 0.47	1.86 ± 0.47	4.14 ± 0.07	1.43 ± 0.25	Metacentric
7	4.20 ± 0.71	2.47 ± 0.32	1.75 ± 0.42	4.09 ± 0.07	1.45 ± 0.22	Metacentric
(7)	4.12 ± 0.72	2.36 ± 0.38	1.77 ± 0.38	4.01 ± 0.10	1.35 ± 0.20	Metacentric
8	4.02 ± 0.69	2.29 ± 0.40	1.72 ± 0.35	3.91 ± 0.08	1.35 ± 0.21	Metacentric
(8)	3.84 ± 0.62	2.09 ± 0.50	1.75 ± 0.22	3.74 ± 0.12	1.31 ± 0.19	Metacentric
9	3.64 ± 0.61	2.06 ± 0.34	1.57 ± 0.29	3.54 ± 0.17	1.33 ± 0.15	Metacentric
(9)	3.48 ± 0.54	1.96 ± 0.31	1.53 ± 0.27	3.40 ± 0.14	1.30 ± 0.19	Metacentric
10	3.35 ± 0.52	1.94 ± 0.33	1.42 ± 0.24	3.27 ± 0.10	1.38 ± 0.19	Metacentric
(10)	3.22 ± 0.46	1.83 ± 0.30	1.40 ± 0.23	3.15 ± 0.13	1.32 ± 0.23	Metacentric
11	3.12 ± 0.46	1.81 ± 0.21	1.31 ± 0.27	3.05 ± 0.12	1.41 ± 0.18	Metacentric
(11)	3.06 ± 0.47	1.83 ± 0.28	1.23 ± 0.20	2.98 ± 0.11	1.50 ± 0.12	Metacentric
12	2.82 ± 0.48	1.63 ± 0.31	1.20 ± 0.19	2.76 ± 0.22	1.36 ± 0.18	Metacentric
(12)	2.64 ± 0.33	1.53 ± 0.20	1.12 ± 0.14	2.59 ± 0.19	1.36 ± 0.13	Metacentric
KL	102.76					

**Table 5:** Fungus-farming ants cytogenetically studied to date and the new reports from the present study. The heterochromatic pattern evidenced by C-band technique, karyotype structure (formula), and genome size is highlighted.

Species Studied	2n (n)	Genome Size 1C (pg)	Local/State	Country	Karyotype	Heterochromatic Pattern (C-Bands)					References
						C	PC	IN	SA	LA	
*Acromyrmex ambiguus*	38	0.33	SP	Uruguay	14 m + 12 sm + 8 st + 4 a (2 m + 6 sm + 16 st + 14 a)	+	+	–	+	–	[28,29]
*Acromyrmex ameliae*	36	-	MG	Brazil	10 m +16 sm + 8 st + 2 a	+	–	–	+	–	[30]
*Acromyrmex aspersus*	38	-	MG	Brazil	8 m + 10 sm + 16 st + 4 a						[31]
*Acromyrmex balzani*	38	0.37	MG	Brazil, French Guiana	12 m + 10 sm + 14 st + 2 a	+	+	–	+	–	[32,33]
*Acromyrmex coronatus*	38 (19)	0.34	MG	Brazil	12 m + 8 sm + 16 st + 2 a	+	+	–	+	–	[32]
*Acromyrmex crassispinus*	38	0.34	MG	Brazil	12 m + 20 sm + 4 st + 2 a						[29,34]
*Acromyrmex disciger*	38	0.33	MG	Brazil	10 m + 12 sm + 14 st + 2 a	+	+	–	+	–	[32]
*Acromyrmex echinatior*	38	0.36		Panama	8 m + 6 sm + 14 st + 10 a	–	–	+	+	–	[32]
*Acromyrmex heyeri*	38	-	RS	Uruguay, Brazil	2 m + 6 sm + 16 st + 14 a						[28,35]
*Acromyrmex hispidus*	38	-		Uruguay	2 m + 6 sm + 16 st + 14 a						[28]
*Acromyrmex lundi*	38 (19)	-	RS	Brazil	10 m + 14 sm + 10 st + 4 a						[29]
*Acromyrmex niger*	38	0.36	MG	Brazil	12 m + 14 sm + 10 st + 2 a	–	+	–	+	–	[32]
*Acromyrmex nigrosetosus*	38(19)	0.35	MG	Brazil	12 m + 14 sm + 10 st + 2 a						[29]
*Acromyrmex rugosus*	38	0.35	MG	Brazil	16 m + 12 sm + 8 st + 2 a	+	+	–	+	–	[32]
*Acromyrmex subterraneus molestans*	38	0.34	MG	Brazil	10 m + 10 sm + 16 st + 2 a						[31,34]
*Acromyrmex subterraneus subterraneus*	38	0.35	MG	Brazil	14 m + 18 sm + 4 st + 2 a	+	+	–	+	–	[29,34]
*Acromyrmex subterraneus brunneus*	38	0.34	MG	Brazil	10 m + 14 sm + 12 st + 2 a	+	–	–	+	–	[30]
*Amoimyrmex striatus*	22	0.35	SC	Brazil	20 m + 2 sm	+	+	–	+	–	[11]
*Amoimyrmex silvestrii*	22			Argentine	20 m + 2 sm						[36]
*Amoimyrmex bruchi*	22			Argentine	20 m + 2 sm						[36]
*Apterostigma madidiense*	(23)	-		Brazil	14 m + 20 sm + 10 st + 2 a						[37]
*Apterostigma madidiense*	24	0.74	MG	Brazil	24 m	+	+	–	–	–	This study
*Apterostigma mayri*	24	-		Panama	24 m	+	–	–	–	–	[38]
*Apterostigma* sp.	20	-		Brazil	6 m + 12 sm + 2 a						[34]
*Apterostigma* sp.	24	-		Panama	24 m						[38]
*Apterostigma* sp.	32	-		French Guiana	14 m + 6 sm + 10 st + 2 t						[39]
*Apterostigma steigeri*	22	-		Brazil	20 m + 2 sm						[37]
*Atta bisphaerica*	22	-	MG	Brazil	12 m + 6 sm + 4 a	+	+	–	–	–	[34,40]
*Atta colombica*	22 (11)	0.31		Panama	12 m + 6 sm + 4 a	+	–	+	–	–	[38]
*Atta laevigata*	22	0.33	MG	Brazil	12 m + 6 sm + 4 a	+	+	–	–	–	[34,40]
*Atta robusta*	22	0.34	ES	Brazil	18 m + 2 sm + 2 st	+	+	–	–	–	[41]
*Atta sexdens*	22	0.33	MG, RS	Brazil	12 m + 6 sm + 4 a	+	+	–	–	–	[34,35,40]
*Atta sexdens*	22	-		French Guiana	18 m + 2 sm + 2 st	+	+	–	–	–	[33]
*Cyphomyrmex cornutus*	22	-		French Guiana	10 m + 12 sm						[39]
*Cyphomyrmex costatus*	20	-		Panama	20 m	+	–	–	–	–	[38]
*Cyphomyrmex rimosus*	32	-		Panama	28 m + 4 a						[38]
*Cyphomyrmex transversus*	24 (12)	-		French Guiana	14 m + 6 sm + 4 a						[33]
*Cyphomyrmex transversus*	42 (21)	0.50	RJ	Brazil	28 m + 14 sm	+	–	–	–	–	This study
*Mycetarotes carinatus*	14	-	MG	Brazil	8 m + 6 sm	+	+	–	–	–	[42]
*Mycetarotes parallelus*	54	0.38	MG	Brazil	26 m + 16 sm + 6 a	+	+	–	+	–	[42]
*Mycetomoellerius fuscus*	18 (9)	0.47	MG	Brazil	16 m + 2 sm	+	+	–	–	–	[43]
*Mycetomoellerius holmgreni*	20 (10)	0.33	MG, SC, RS	Brazil	20 m	+	+	–	–	–	[7]
*Mycetomoellerius iheringi*	20 (10)	0.40	SC	Brazil	18 m + 2 sm	+	+	–	–	–	[44]
*Mycetomoellerius relictus*	20 (10)	-	MG	Brazil	20 m	+	+	–	–	–	[37]
*Mycetomoellerius* sp.	22	-	MG	Brazil	18 m + 4 sm						[37]
*Mycetophylax conformis*	30 (15)	0.31	RJ, SP	Brazil	22 m + 8 sm	+	+	–	+	–	[10]
*Mycetophylax morschi*	30 (15)	0.34	RJ, RS, SC	Brazil	18 m + 6 sm + 2 a	+	–	–	–	–	[10]
*Mycetophylax morschi*	26 (13)	0.31	SC	Brazil	18 m + 10 sm + 2 a	+	–	–	–	–	[10]
*Mycetophylax morschi*	28 (14)	-	BA	Brazil	18 m + 10 sm	+	–	–	–	–	[17]
*Mycetophylax simplex*	36 (18)	0.41	SC, PR, SP	Brazil	20 m + 16 sm	+	+	–	+	–	[10]
*Mycocepurus goeldii*	8	-	MG	Brazil	8 m	+	+	–	–	–	[45]
*Mycocepurus goeldii*	8 (4)	0.42	SC	Brazil	4 m + 4 sm						This study
*Mycocepurus* sp.	8	-		Panama	4 m						[38]
*Myrmicocrypta* sp.	30	-		French Guiana	22 m + 2 sm + 6 a						[33]
*Myrmicocrypta* sp.	28 (14)	0.48	RJ	Brazil	24 m + 4 sm	+	+	–	–	–	This study
*Sericomyrmex amabilis*	50	0.45		Panama	50 m	+	+	–	–	–	[38]
*Sericomyrmex* sp.	50 (25)	-	MG	Brazil	44 m + 6 sm						[37]
*Serycomyrmex parvulus*	50 (25)	0.42	MG	Brazil	30 m + 14 sm + 6 st	+	+	–	–	–	This study
*Trachymyrmex septentrionalis*	20 (10)	0.25		Panama	20 m						[38]
*Trachymyrmex* sp.1	12 (6)	-		Panama	12 m	+	–	+	–	–	[38]
*Trachymyrmex* sp.2	18	-		Panama	18 m						[38]

## Data Availability

All data are referenced and available in the manuscript.

## Supplementary Materials

The following are available online at https://www.mdpi.com/article/10.3390/insects12121084/s1, Table S1: Models of molecular evolution by genes and codons implemented in the Bayesian analyses to infer the molecular phylogeny of fungus-growing ants. Figure S1: Chronogram of fungus-farming ants estimated by fossilized birth-death (FDB) model based on five molecular markers (see Materials and Methods).
