# Supplementary material for: Karyotype Diversity, Mode, and Tempo of the Chromosomal Evolution of Attina (Formicidae: Myrmicinae: Attini): Is There an Upper Limit to Chromosome Number?
Source: Insects. 2021 Dec 2;12(12):1084. doi: 10.3390/insects12121084 (PMC8707115; doi:10.3390/insects12121084)
Supplement: Supplementary file 1 [file insects-12-01084-s001.zip › tableS1.pdf]

Table S1: Models of molecular evolution by genes and codons

Gene (number of base pairs)	Position	Model
wingless	1st – first position	TRN+I
	2nd – second position	TRN+I
	3rd – third position	TRN+G
***elongation factor*-1 *alpha* F1**	1st – first position	TRN+I
	2nd – second position	HKY+I
	3rd – third position	TRN+G
***elongation factor*-1 *alpha* F2**	1st – first position	TRN+I
	2nd – second position	HKY+I
	3rd – third position	TRN+G
long-wavelength rhodopsin	1st – first position	HKY+I
	2nd – second position	F81+I
	3rd – third position	TRN+G
topoisomerase I	1st – first position	TRN+I
	2nd – second position	HKY+I
	3rd – third position	HKY+I
