# Supplementary figures and images for: Karyotype Diversity, Mode, and Tempo of the Chromosomal Evolution of Attina (Formicidae: Myrmicinae: Attini): Is There an Upper Limit to Chromosome Number?
Source: Insects. 2021 Dec 2;12(12):1084. doi: 10.3390/insects12121084 (PMC8707115; doi:10.3390/insects12121084)

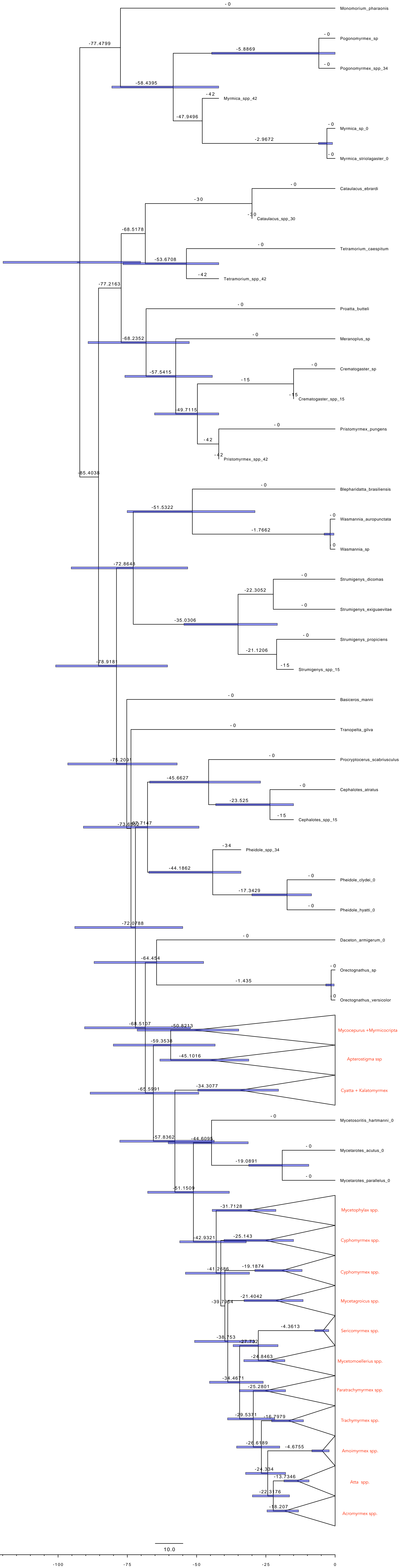

Supplement: Supplementary file 1 [file insects-12-01084-s001.zip › FigS1.pdf]
